# Co-prevalence of SARS-CoV-2 and Noroviruses in Wastewater Collection and Treatment Facilities of the Eastern Cape Region, South Africa: An Exacerbated Public and Environmental Health Risk Concern

**DOI:** 10.1007/s12560-026-09688-0

**Published:** 2026-04-20

**Authors:** Mbasa Dlamini, Luyanda Msolo, Nolonwabo Nontongana, Kingsley Ehi Ebomah, Anthony Ifeanyi Okoh

**Affiliations:** 1 SAMRC Microbial Water Quality Monitoring Centre, University of Fort Hare, Alice, 5700 South Africa; 2 DSTI-NRF SARChI in Water Quality and Environmental Genomics, University of Fort Hare, Alice, 5700 South Africa

**Keywords:** Influent, Wastewater treatment facilities, SARS-CoV-2, Norovirus GI, Norovirus GII, RT-qPCR

## Abstract

Severe acute respiratory syndrome Coronavirus-2, Norovirus GI and GII have been identified as one of the leading primary agents of lethal diseases such as COVID-19 and acute gastroenteritis, causing critical problems in public health, particularly in children, the elderly, and individuals with weakened immune systems. These pathogens contribute to substantial morbidity, mortality and pose a serious threat to human health, imposing a tremendous economic burden globally. Hence, this study sought to profile the co-occurrence of SARS-CoV-2 and Noroviruses in wastewater collection and treatment facilities within Buffalo City and Amathole District regions, Eastern Cape Province, South Africa. Raw wastewater samples were collected from the wastewater treatment facilities on a weekly basis for a 6-month sampling regime using the grab sampling technique. Total Ribonucleic acids (RNA) were extracted and purified using the commercial Total RNA extraction kits and the extracted RNA sample were further profiled for the presence of SARS-CoV-2 RNA and Noroviruses GI and GII using RT-qPCR. A total of 213 samples were screened using RT-qPCR. SARS-CoV-2 was detected in 146 (69%) samples, Norovirus GI in 58 (27%), Norovirus GII in 25 (12%) and 25 (12%) samples showed co-infection with both GI and GII norovirus targets. The RNA concentration of the extracted samples from wastewater treatment facilities in this study ranged between 102 ng/µl and 12,828 ng/µl. It was observed that the SARS-CoV-2 GC/mL range from 5.22 × 10^2^ to an extreme 2.5650 × 10^4^ GC/mL. The study findings demonstrate a high prevalence of SARS-CoV-2 RNA in wastewater, affirming the utility of wastewater surveillance as an early warning system for potential outbreaks at community levels. Furthermore, the study showed the co-occurrence of SARS-CoV-2 alongside Norovirus GI and GII in wastewater milieu, highlighting an exacerbated potential risk of co-infection and a major threat to public health and environmental safety.

## Introduction

Despite significant advances in science, infections and disease outbreaks remain critical global health concerns affecting both individuals and communities. Millions of people have died and been burdened by this issue throughout human history (Dye, 2014). Viral diseases caused by the occurrence of SARS-CoV-2, Norovirus Genogroup I and II in wastewater are a major source of morbidity and death globally (Hoque et al., 2023). Waterborne diseases are caused globally by the introduction of millions of human pathogenic virus particles into wastewater from infected individuals (Okoh et al., 2010). These microbes can be transmitted by both direct touch and unintentional aerosol ingestion or inhalation (Lanrewaju et al., 2022). Therefore, wastewater-based epidemiology provides a quick and cost-effective alternative for monitoring SARS-CoV-2 and other harmful viruses.

Coronavirus Disease 2019 is currently ranked amongst the serious and urgent global health concerns, causing illness and death while also contributing to significant harm to economic and societal wellbeing (Panneer et al., 2022). It is a serious respiratory disease that affects people and is caused by SARS-CoV-2 (Bojkova et al., 2020; Yan et al., 2020). Coronaviruses are members of the *Coronaviridae* family, and are enclosed, positive single-stranded RNA viruses (Jain & Barhate, 2020). SARS-CoV-2 are mostly transmitted by respiratory droplets and aerosols that are produced during sneezing or coughing and through direct contact. Therefore, it is essential for disease management procedures to promptly identify and monitor this virus, which is a serious public health risk. Although SARS-CoV-2 and Noroviruses are members of different families and cause different diseases such acute gastroenteritis and COVID-19, their modes of transmission are similar. For instance, both viruses are extremely infectious and spread through close contact with infected individuals or objects. The symptoms of SARS-CoV-2 and NoV infections are quite similar, including diarrhea, vomiting, and nausea (Menni et al., 2022; Vihta et al., 2023).

SARS-CoV-2 is the virus responsible for COVID-19 and over 6 million people have died as a result of COVID-19, which has had a devastating impact globally (Msemburi et al., 2023). The SARS-CoV-2 pandemic has historically significant effects on society and public health (Koella et al., 2022). This is an illness that may afflict people of any age. However, those with medical conditions and those over 60 of age are more likely to get severe COVID-19 infection (Gao et al., 2021). Globally, the COVID-19 pandemic caused over 362,705 deaths and 5,817,385 reported cases, including 1,761,503 total reported cases and 103,700 fatalities (Bhandari et al., 2021). The high COVID-19 incidence highlighted attention to the shortcomings of conventional public health case surveillance methods in providing real-time intelligence, underscoring the necessity of ongoing innovation and modernization. Notwithstanding its limitations such as lack of accuracy or insufficient data, nationwide COVID-19 case surveillance is essential (Ibrahim, 2020).

Nevertheless, Noroviruses are the primary cause of non-bacterial acute gastroenteritis in humans globally and are excreted in high concentration in human feces (Hall et al., 2013). They are commonly known to spread through a variety of routes, such as contaminated food and water (Ngazo et al., 2008; Chhabra et al., 2019). The main infection targets for NoV-GI and NoV-GII are school-age children and adults greater than or equal to 65 years of age (Rhoca-Pereica et al., 2018). Noroviruses, especially those belonging to genogroups I and II, have drawn significant amount of interest in the field of public health. Although the intensity of norovirus infections varies, they frequently cause a devastating combination of diarrhea, vomiting, and stomach discomfort (Brown et al., 2017). The need for effective and early detection methods has never been more paramount due to its fast spread.

The harmonized wastewater-based surveillance (WBS) is a very useful method for assessing the caseload and detecting infectious disease. It provides a complementary tool, and sometimes primary for assessing illness prevalence and early outbreak warning. This was especially noticeable during the COVID-19 outbreak. SARS-CoV-2 RNA monitoring in wastewater has demonstrated significant potential in identifying the virus prior to the occurrence of cases (Ahmed et al., 2021; Claro et al., 2021). According to the most recent studies, WBE may serve as an early warning indicator of potential disease outbreaks within a community (Kitajimi et al., 2020; Orive et al., 2020). Such WBE is especially crucial for analyzing data retrospectively in order to estimate the likely population impacted by the infection, due to the possibility that asymptomatic individuals who are not properly identified by clinical monitoring may potentially transmit the virus through their feces.

In certain parts of the world, SARS-CoV-2, norovirus GI and GII disseminate in wastewater via human waste and have been linked to disease outbreaks due to the introduction of feces on the environment. However, there are few documented reports on the prevalence of these viral pathogens within the Eastern Cape region. As a result, this is the first study sought to evaluate the co-occurrence of these pathogens in wastewater influents collected from selected wastewater treatment facilities in the Eastern Cape region, South Africa.

## Materials and Methods

### Description of the Study Area

**Fig. 1:**
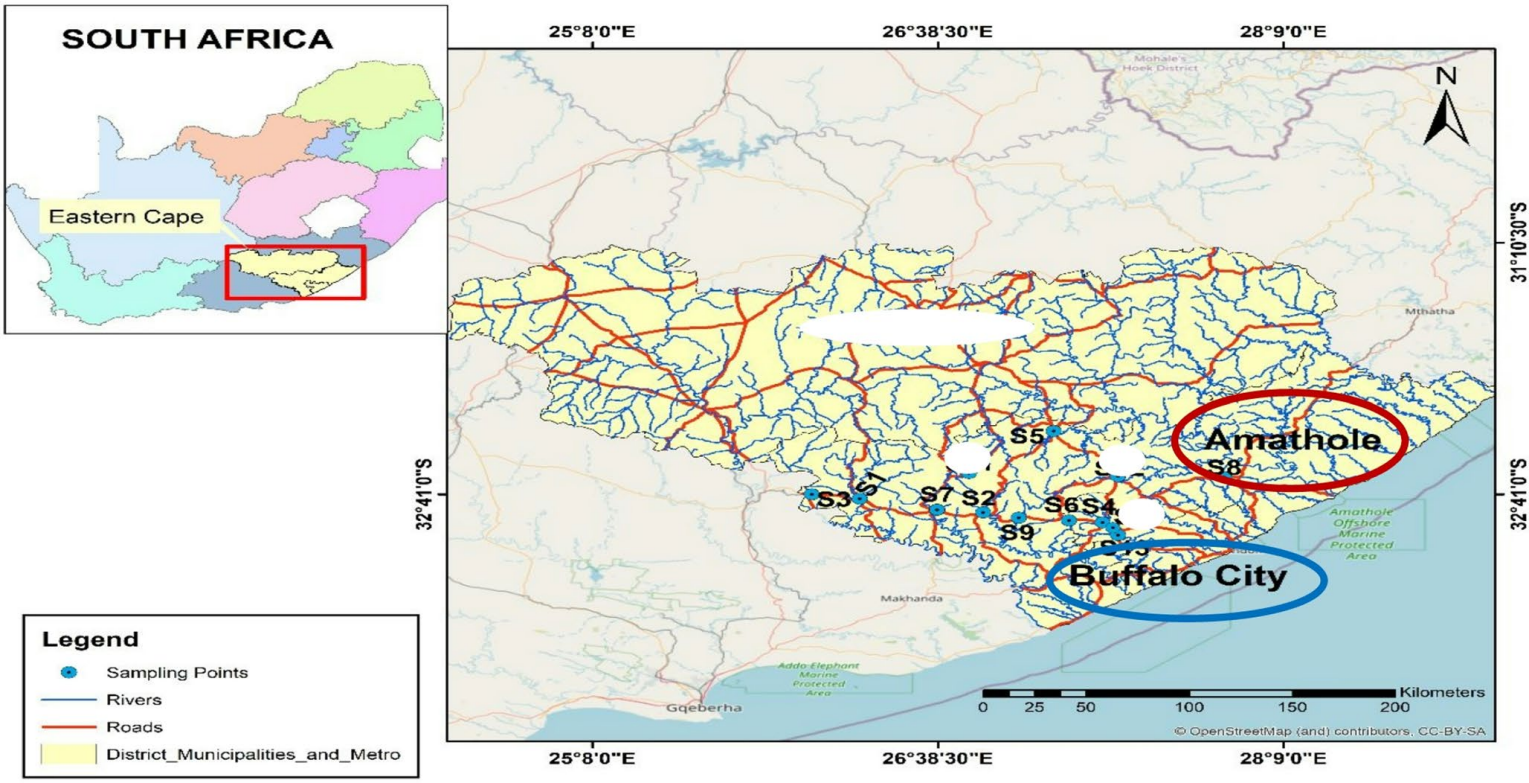
Map of the Eastern Cape Province showing the geographical appearance and location of Buffalo City Municipality and Amathole District where the study was carried

This study was carried out in selected wastewater treatment plants located in the Amathole District and Buffalo City Municipalities (Fig. 1). The Amathole District Municipality (ADM) is a Category C municipality located in the Eastern Cape’s central region. The municipality consists of six local municipalities such as Mnquma, Amahlathi, Raymond Mhlaba Mbhashe, Great Kei and Ngqushwa (Amathole District & Municipality, 2020). The Buffalo City Metropolitan Municipality is situated on the east coast and is classified as a Category A municipality which is a municipality that has exclusive municipal executive and legislative authority in its area. It is comprised of an urban corridor that extends from East London to the east, to Mdantsane, and to Dimbaza in the west. It also includes a wide area of rural communities on both sides of the urban corridor particularly in regions such as Macleantown, Sandisiwe, and areas surrounding Mdantsane and Chalumna (Buffalo City & Municipality, 2019). Amathole District and Buffalo City Municipalities consist of several Wastewater Treatment Plants (WWTPs) and these WWTPs differ in catchment area, treatment processes, population density and design capacity (Table 1). In the Amathole District and Buffalo City Municipalities, a network of facilities serves as the delivery system for healthcare services. In all, these areas accommodate 236 clinics and 8 Community Health Centres that operate 24-hour services (Eastern Cape Department of Health, 2025).

**Table 1:** Population density, catchment areas, and technology use of the study sites (Eastern Cape Development Corporations, 2019)

Site(s)	Capacity of the Plant (Megaliters/Day)	Technology Use of Wastewater Treatment Plants	Area (Coverage)	Population Density	Population	Catchment Area	Population Equivalent
ADE	0.4	Activated sludge model	40.0 km^2^	300/km^2^	12,191	Koonap river	12,191 p.e.
AL	2	Activated sludge model	9.85 km^2^	1536/km^2^	15,143	Tyume river	15,143 p.e.
BED	0.5	Oxidation ponds	14.6 km^2^	600/km^2^	8,770	Nyarha river	8,770 p.e.
BIS	2	Stabilization pond	8.082 km^2^	1400/km^2^	11,192	Yellowwoods river	11,192 p.e.
DIM	8	Activated sludge model	17.289 km^2^	1300/km^2^	21,783	Mdizeni stream	21,783 p.e.
FBA	3.0	Sludge activation and sludge dryer beds	82.81 km^2^	310/km^2^	25,668	Kat river	25,668 p.e.
SCH	7	Activated sludge model	65.52 km^2^	520/km^2^	34,019	Buffalo river	34,019 p.e.
SEY	12.3	Sludge activation and sludge dryer beds	2.59 km^2^	950/km^2^	2,467	Gesie River	2,467 p.e.
ZWE	7.5	Activated sludge model, Bio-filters	4.646 km^2^	3900/km^2^	18,189	Buffalo river	18,189 p.e.

### Sample Collection

Raw wastewater samples were collected from nine wastewater treatment plants referred to as ADE, AL, BED, BIS, DIM, FBA, SCH, SEY and ZWE, as shown in Table 2. Pre-sterilized 500 mL sampling bottles and TeleScoop water sampler were used to collect raw wastewater samples from various wastewater treatment facilities on a weekly basis for a 6-months sampling regime using the grab sampling technique. The obtained samples were properly transported in ice-filled cooler boxes to the University of Fort Hare’s SAMRC Microbial Water Quality Monitoring Laboratory for analysis within a few hours of collection. Strict safety and handling measures were adhered to. Personal Protective Equipment (the appropriate PPE were always worn, including gloves, eye protection, resistant clothing and masks) and alcohol-based hand sanitizers were used during wastewater sample collection and handling.

**Table 2:** A summarized distribution of SARS CoV-2 RNA obtained across the study sites throughout the sampling regime

Sampling point (WWTP)	Number of samples retrieved from the sites	Number of positive samples	Samples positive for SARS CoV-2 genomes (%)
ADE	25	10	40
AL	20	12	60
BED	15	10	67
BIS	35	30	86
DIM	26	20	77
FBA	20	15	75
SCH	26	19	73
SEY	20	10	50
ZWE	26	20	77

A total of 146 (69%) samples out of 213 samples possessed the nucleocapsid gene of SARS-CoV-2 virus

### Total RNA Extraction and Quantification

Total RNA was extracted from the collected wastewater samples following the optimized extraction protocol as outlined by Johnson et al. (2021). Briefly, about 200 mL aliquots of homogenized untreated wastewater samples were centrifuged for 20 min at 2500 × g in sterile centrifuge tubes. The supernatant was disposed of and about 7.5 mL of the obtained pellet was used to extract total RNA using the QIAGEN RNeasy PowerSoil Total RNA Kit following the manufacturer’s instructions. The Nanodrop™ One Microvolume UV-Vis spectrophotometer was used to determine the concentration and ensure purity of the extracted total RNA.

### Profiling of SARS-CoV-2 Genomes by RT-qPCR

The QuantStudio 5 qPCR system was used to detect and quantify SARS-CoV-2 genomes from the extracted total RNA samples using the iTaq Probe-Based qPCR assay (BIO-RAD, USA). SARS-CoV-2 RNA was amplified and quantified in the processed samples by amplifying the SARS-CoV-2_N1-P and SARS-CoV-2_N2-P target genes of the Nucleocapsid (CDC, 2020; Johnson et al., 2021; Street et al., 2021). Table 3 outlines the optimized PCR cycling conditions that were used for the identification and quantification of SARS-CoV-2 RNA across the study sites. Reverse transcription was carried at 95℃ for 10 min, followed by Polymerase Activation and DNA denaturation at 95℃ for 3 min, denaturation at 95 ℃ for 15 s and annealing/extension at 60℃ for 1 min. Amplification consisted of 40 cycles.

**Table 3:** Optimized PCR cycling conditions that were used for the detection and quantification of SARS-CoV-2 RNA across the study sites

Real Time PCR System	Settings	Steps	Time	Temperature (°C)	Cycles
Quant-Studio 5 PCR machine	Fast/Standard	Reverse transcription	10 min	95	40
		Polymerase Activation and DNA Denaturation	3 min	95	
		Denaturation	15 s	95	
		Annealing/Extension (Data collection)	1 min	60	

### Detection of Noroviruses by Real Time PCR

The detection of Noroviruses from the processed total RNA samples was done using the Viasure Norovirus GI + GII Real Time PCR Detection Kit following manufacturer’s guidelines. Different reagents or material were used such as rehydration buffer (15µ), Norovirus positive and negative control (5 µl, where positive control was supplied as non-infectious synthetic lyophilized cDNA and nuclease free water was used as non-template negative control), nuclease free water. The thermal cycling conditions utilized for the detection of Noroviruses are shown in Table 4 and reverse transcription was carried at 45℃ for 15 min, followed by initial denaturation at 95℃ for 2 min, denaturation at 95℃ for 10 s and annealing/extension at 60℃ for 50 s.

**Table 4:** Optimized thermal cycling conditions for the detection of norovirus GI and GII (CerTest Biotec, 2024)

Cycles	Steps	Time	Temperature (°C)
1	Reverse transcription	15 min	45
1	Initial Denaturation	2 min	95
45	Denaturation	10 s	95
	Annealing/Extension (Data collection)	50 s	60

## Results and Discussion

**Fig. 2:**
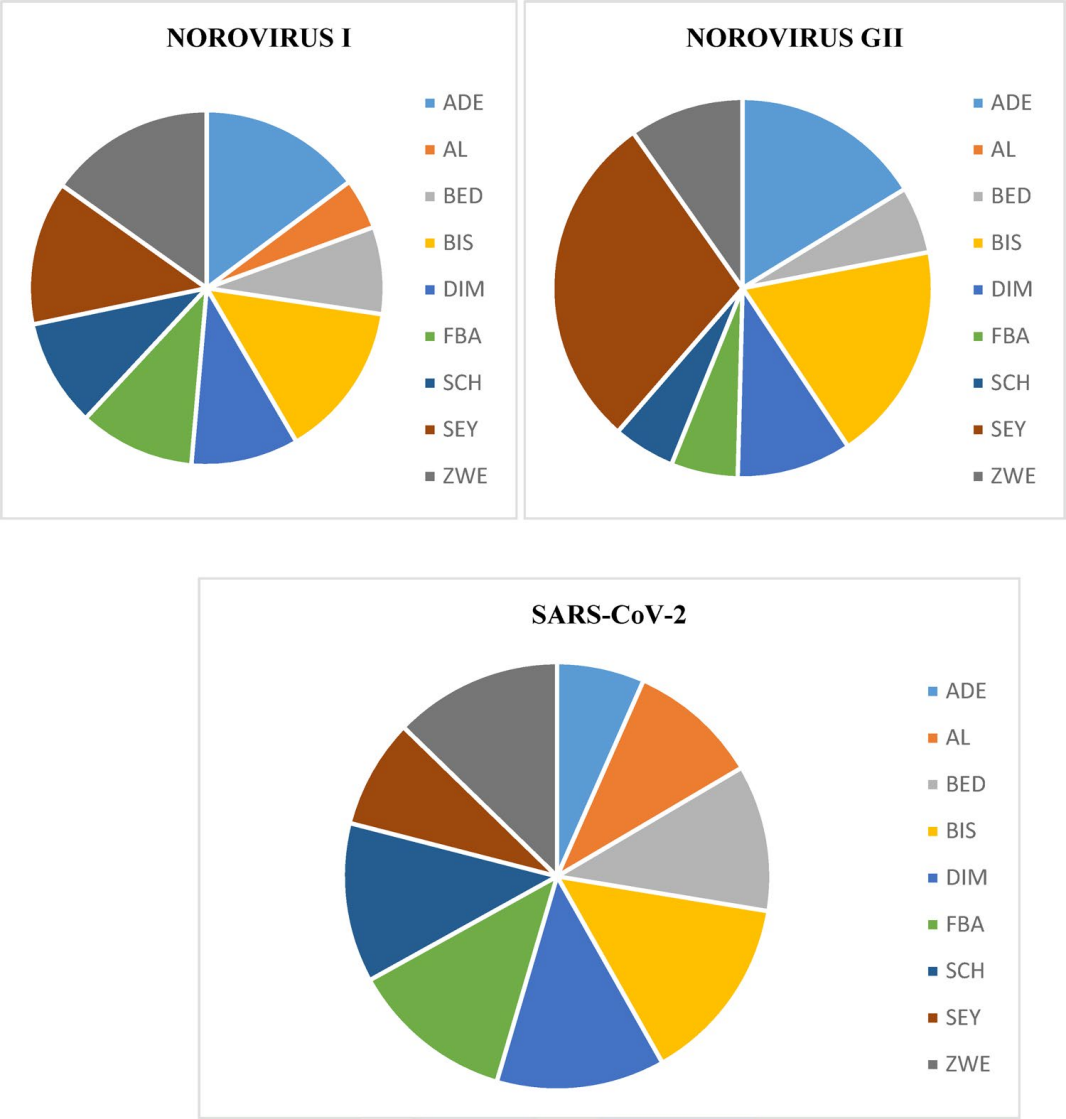
Representation of the overall distribution of the panel of viral pathogens obtained from the study sites throughout the sampling period

A total of 213 samples were collected from selected wastewater treatment facilities and screened using RT-qPCR. A total of 146 (69%) samples were positive for SARS-CoV-2 of which a sum of 25 (12%) total RNA samples were positive for Norovirus GI and GII targets (Fig. 2; Table 5). There was a trend in the total RNA concentrations across the different sampling sites, week 38 site ZWE recorded the highest total RNA concentration of 12.828 × 10^3^ ng/µl. It was observed that the SARS-CoV-2 GC/mL range from 5.22 × 10^2^ to an extreme 2.5650 × 10^4^ GC/mL and Table 2 outlines the summary of the obtained SARS CoV-2 from wastewater samples.

**Table 5:** Overall summary of the distribution of the panel of viral pathogens obtained in the study period

	SARS-CoV-2	Noroviruses			No. of positive samples for a one/combination of NoV’s GI/GII
		Norovirus GI	Norovirus GII	Norovirus GI and GII	
No. of positive samples	146 (69%)	58 (27%)	25 (12%)	25 (12%)	108 (51%)

In the present study, 146 (69%) samples were positive for SARS-CoV-2. Fifty-eight (27%) samples were confirmed as positive for Norovirus GI and 25 (12%) samples were positive for Norovirus GII and 25 (12%) samples were positive for both GI and GII Norovirus

From a total of 146 samples which were positive for SARS-CoV-2, 108 samples were also positive for one or a combination of Norovirus GI/GII targets

### Profiles of SARS-CoV-2 by Real Time-PCR

**Fig. 3:**
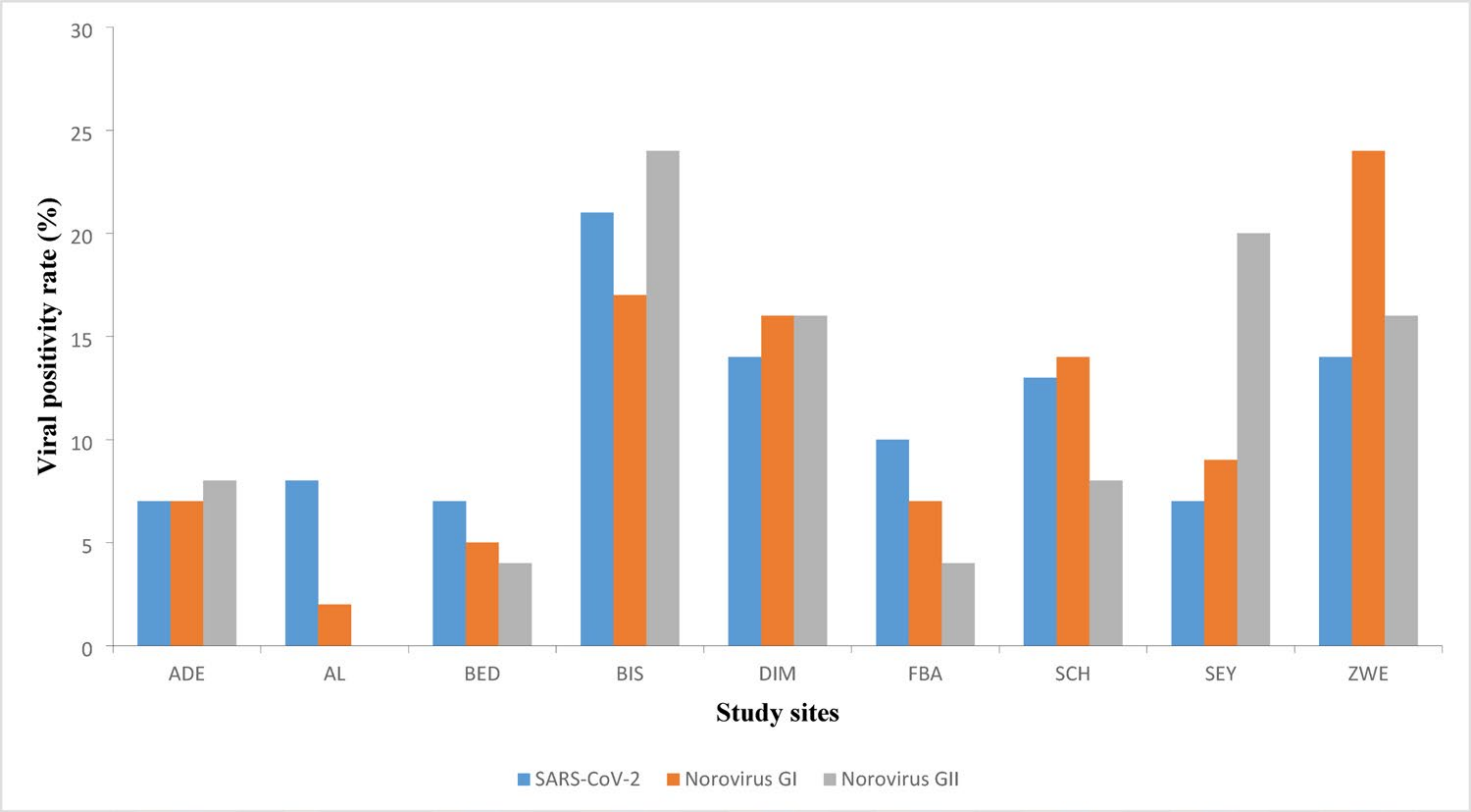
Graphical representation of the viral positivity rates obtained from the study sites throughout the sampling period

It was found that the genome copies of SARS CoV-2 were ranging from 5.22 × 10^2^ to an extreme 2.5650 × 10^4^ GC/mL. Relatively high genome copies of approximately 2.5650 × 10^4^ GC/mL were observed from the BIS site in week 33 whereas low viral genome copies (5.22 × 10^2^) were observed from the DIM site in week 30 (see Fig. 3).

### Profiles of Norovirus GI and GII by Real Time-PCR

A total of 213 samples were screened for the presence of Norovirus targets using RT-qPCR. Fifty-eight (27%) samples were positive for Norovirus GI, 25 (12%) samples were confirmed positive for Norovirus GII and 25 (12%) samples were positive for both GI and GII Norovirus targets (Tables 6 and 7).

**Table 6:** Profiles of Norovirus Genogroup I and II from wastewater samples collected in selected wastewater treatment facilities

Sampling point (WWTP)	Number of samples screened for NoV’s from each site (*n* = 213)	Number of positive samples for NoV’s		Number of positive samples for the combination of Norovirus GI and GII	% of positive NoV’s	
		Norovirus GI	Norovirus GII	Norovirus GI and GII	Norovirus GI	Norovirus GII
ADE	5	4	2	2	80	40
AL	4	1	0	0	25	0
BED	7	3	1	1	43	14
BIS	13	10	6	6	77	46
DIM	17	9	4	4	53	24
FBA	7	4	1	1	57	14
SCH	15	8	2	2	53	13
SEY	7	5	5	5	71	71
ZWE	17	14	4	4	82	24
TOTAL	92	58	25	25		

**Table 7:** The summary on the distribution of Norovirus Genogroup I and II from wastewater samples collected in selected wastewater treatment facilities

Number of screened samples (*n* = 213)	Number of positive Norovirus GI samples (*n* = 213)	Number of positive Norovirus GII samples (*n* = 213)	Number of positive Norovirus for both GI and GII samples (*n* = 213)
	% of positive Norovirus GI **58** (**27%)**	% of positive Norovirus GII **25 (12%)**	% of positive Norovirus GI and GII **25 (12)%**

The role of wastewater as a vehicle for transmission of SARS-CoV-2 along with Norovirus Genogroup I and II is well documented, but limited information is available or published in some parts of the world. There are still data gaps also in the proliferation of these pathogens in wastewater facilities in some regions of South Africa, particularly in the Eastern Cape Province, henceforth the study aimed at determining the co-occurrence of these pathogens in wastewater milieu, in order to assess the associated communal health risks within the Amathole and Buffalo City District Municipalities.

The RNA concentration of the extracted samples from Amathole and Buffalo City District wastewater treatment facilities ranged between 102 ng/µl and 12,828 ng/µl. The observation of high RNA in wastewater influents cause significant issues in public health. For instance, the high RNA concentrations obtained in the present study may be linked to the existence of non-viral RNA sources such as microbial communities, plant debris, or extracellular RNA produced by human and animal cells (Lever et al., 2015). The prevalence of Ribonucleic acids in wastewater samples corroborates the findings reported by several researchers (Osuolale & Okoh, 2017; Wang et al., 2020; Rothman et al., 2021). Thus, our findings affirm the viral indicators in wastewater environments and imply that newly infected people contribute substantial viral loads to wastewater, with the majority of this shedding potentially taking place early in the infection, before the person seeks medical attention and is tested (Shah et al., 2022). According to a recent study by Qongwe et al. (2024), fecal oral route of SARS-CoV-2 is known to occur in developing countries with inadequate sanitation, implementing surface waters and sewage notable SARS-CoV-2 reservoirs. Similarly, the higher quantities of total RNA detected from the different sites in the present study further emphasize the concept highlighted by Haramoto et al. (2020). The concept highlighted is that higher quantities of total RNA in wastewater influents indicate increased existence of non-viral RNA sources such as microbial communities and plant debris. This further represent wastewater treatment facilities as a source of viral contamination.

This study assessed the distribution and prevalence of SARS-CoV-2 genomes in wastewater samples. The results of this study showed that the genome copies of SARS CoV-2 were ranging from 5.22 × 10^2^ to 2.5650 × 10^4^ GC/mL. Johnson et al. (2021) carried out a study on detection of SARS-CoV-2 RNA in untreated wastewater. The obtained genome copies ranged between 4.6 × 10^3^ and 454 × 10^3^ GC/mL which were much greater than the one found in the present study. Moreover, the study by Tanhaei et al. (2021) showed less genome copies than the present study. The results of this study demonstrate a high prevalence of SARS-CoV-2 genomes in wastewater, highlighting the potential risk of contracting COVID-19, a devastating human health burden. Numerous studies conducted globally recorded the presence of SARS-CoV-2 in wastewater milieu, highlighting the necessity of a worldwide WBE approach to monitor the dynamics of viral transmission (Ahmed et al., 2020; Medama et al., 2020; Lodder & Roda, 2020).

Noroviruses are thought to be the primary cause of acute nonbacterial gastroenteritis in people of all ages globally (Liao et al., 2021). In the current study, wastewater samples were analyzed to compare the positive rates and Cq values were used as indication of the positive amplification of targets fragments and notably only samples with Cq values below 40 (cut-off Cq value) were considered positive for Norovirus, based on the established threshold. The lowest Cq value was obtained in week 10 site BED whereas the highest Cq value was obtained in week 26 site BIS and these results were found in Norovirus GI.

The findings reported from other studies revealed a high occurrence of Norovirus GI and GII in wastewater samples (Zhou et al., 2016; Huang et al., 2022) which corroborates the findings of this study. This therefore highlights the need for continued wastewater monitoring models to advocate proactive or preventative strategies against potential Norovirus outbreaks that may impose adverse effects on public and environmental health (Jacqueline et al., 2022). Moreover, increased viral loads in wastewater signal ongoing transmission within the community, necessitating public health countermeasures to circumvent the spread of the virus which may have dire public health and socio-economic effects. The detection of human noroviruses in wastewater matrices have been documented in numerous studies (Okoh et al.2010; Teixeira et al., 2020; Stobnicka et al., 2022; Alex et al., 2023), thus indicating a high prevalence of these pathogens.

The impacts of malfunctional wastewater treatment plants, environmental settings, and the lack of proper sanitation are a cause for concern in South Africa as they act as an important vector for transmission of pathogenic microorganisms such as SARS CoV-2 and Noroviruses. Several studies into South Africa’s water quality have revealed that this problem results in the pollution of water sources, which are necessary for the majority of rural residents’ household and other needs (Edokpayi et al., 2017). Multiple research studies have shown that wastewater from treatment facilities is a significant source of pollution, contributing to a variety of contaminants detected in water resources (Carey & Migliaccio, 2009; Deblonde et al., 2011; Naidoo & Olaniran, 2014). Certain point-sources release municipal raw water, which is then directed toward receiving waterbodies such as ponds, rivers, lakes, streams and groundwater. Problems with point-source pollution not only significantly raise treatment costs (Wato et al., 2020), but they additionally introduce a variety of potentially infectious organisms into the water that many rural populations may receive, increasing the risk of waterborne illnesses caused by SARS-CoV-2 and Noroviruses. Currently, there is no proof to suggest that co-infection with SARS-CoV-2 and Noroviruses leads to chronic or severe conditions other than respiratory or gastroenteritis symptoms. Therefore, addressing infrastructure, ensuring proper sanitation and improving environmental conditions are critical measures to mitigate the dissemination of SARS-CoV-2 and Noroviruses in the environment.

## Conclusion and Recommendations

The findings of this study demonstrate a high co-occurrence of SARS-CoV-2, Norovirus GI and GII disseminating in the wastewater milieu of the BCM and ADM municipal regions. This high prevalence is indicative of an exacerbated potential risk of contracting diseases associated with consumption of inadequately treated or untreated wastewater. Moreover, the poorly maintained WWTPs in the Eastern Cape Province remain a concern for environmental and human health. Therefore, understanding virus prevalence, molecular epidemiology, viability, and circulation through surveillance studies and evaluating the safety of wastewaters is essential for reducing the burden of diseases. Thus, it is crucial that individuals should follow precautions in order to prevent potential infections of SARS-CoV-2 and Noroviruses which causes diseases in humans and death. Therefore, further studies on the assessment of the quality of effluents and the associated receiving watershed are required to monitor the presence of these pathogenic microorganisms that are detected in association with wastewater milieu in the region. There is an urgent need for the development and application of innovative wastewater collection and treatment approaches to alleviate the lingering plight of pathogenic microbes in water and wastewater matrices, to improve public health and environmental safety.

## Data Availability

All relevant data are within the paper.
